# Treatment, Outcome, and Relapse of Spontaneous and Nonspontaneous Cerebrospinal Fluid Leak

**DOI:** 10.3390/brainsci12030340

**Published:** 2022-03-02

**Authors:** Yi-Cheng Tai, Yi-Sheng Tai, Chang-Hsien Ou, Chun-Chung Lui, Hao-Kuang Wang, Hung-Chang Kuo, Shih-Pin Hsu

**Affiliations:** 1 Department of Neurology, E-DA Hospital/I-Shou University, Kaohsiung 824, Taiwan; b88401074@ntu.edu.tw; 2 Department of Urology, Fu Jen Catholic University Hospital, New Taipei 243, Taiwan; taiyisheng@gmail.com; 3 Institute of Occupational Medicine and Industrial Hygiene, National Taiwan University, Taipei 100, Taiwan; 4 Department of Radiology, E-DA Hospital/I-Shou University, Kaohsiung 824, Taiwan; ed104559@edah.org.tw; 5 Division of Medical Image, E-DA Cancer Hospital/I-Shou University, Kaohsiung 824, Taiwan; ed109491@edah.org.tw; 6 Department of Neurosurgery, E-DA Hospital/I-Shou University, Kaohsiung 824, Taiwan; ed101393@edah.org.tw; 7 Department of Neurology, E-DA Cancer Hospital/I-Shou University, Kaohsiung 824, Taiwan; ed100256@edah.org.tw; 8 School of Medicine, I-Shou University, Kaohsiung 824, Taiwan

**Keywords:** cerebrospinal fluid, CSF, leak, blood patch, spontaneous

## Abstract

Cerebrospinal fluid (CSF) leak can be spontaneous or nonspontaneous. The management options include conservative treatments, blood patch, and surgical repairs. We compared clinical symptoms, image findings, management options, hospitalization, and relapse rates among different causes of CSF leaks. Eighty-one patients were recruited: 20 with spontaneous and 61 with nonspontaneous CSF leaks. Nonspontaneous causes included lumbar puncture, surgery, and trauma. Surgery sites comprised sphenoid, spine, skull base, and calvaria. Spontaneous CSF leak came from the sphenoid or spine. Age, gender, body mass index, initial symptoms, hospitalization, treatment courses, and recurrence rates showed no difference between the groups. The spontaneous group had higher CSF accumulations on their MRIs. MRI pachymeninge enhancement showed the highest sensitivity (78.6%) for intracranial hypotension. Meningitis occurred in 1/3 of sphenoid, skull base, and calvarian surgeries. Earlier reoperation was correlated with shorter hospitalization (r = 0.651), but the recurrence rates were similar. Longer intervals between surgery and CSF leak encouraged reoperation. Among the spontaneous spine and lumbar puncture-related CSF leaks, 57.1% of them responded to 4 days of conservative treatment. Among the trauma-related CSF leaks, 90.9% of them required surgical repair. The demographic data and symptoms were similar in various groups of CSF leak. The symptom onset durations and treatment strategies were different. However, the recurrence rates were similar.

## 1. Introduction

Cerebrospinal fluid (CSF) leak can be spontaneous (primary) or nonspontaneous (secondary) [1]. Clinical symptoms include neck pain, tinnitus, changes in hearing, photophobia, and/or nausea. Headache attributed to low CSF pressure, also known as low-pressure headache, is caused by CSF leak or reduced CSF production [2]. Spontaneous CSF leak is commonly found in the sphenoid or in the spine. Procedure-related CSF leak includes lumbar puncture [3]; transsphenoidal surgery [4]; vestibular schwannoma surgery [5]; and cervical [6], thoracic [7], and lumbar spine surgeries [8]. Most literature focused on the incidence and prevention of CSF leak during the procedure. Data associated with the management or incidence of relapse were lacking. In addition, there was no literature comparing the different causes of CSF leak. Thus, we conducted this retrospective study to assess the onset duration, clinical symptoms, management, lengths of hospitalization, and relapse of spontaneous and nonspontaneous CSF leak, and analyzed the data between the two to provide a better treatment strategy.

## 2. Materials and Methods

We reviewed medical charts from August 2004 to August 2021, and recruited patients diagnosed with CSF leak and headache attributed to low CSF pressure based on *The International Classification of Headache Disorders*, 3rd edition [2]. We excluded patients with minor clinical symptoms that did not require hospitalization. Patients without clinical or radiological evidence of CSF leak or without temporal attribution to presumed causes (for example, operation of the cervical spine with CSF leak on the lumbar spine or traumatic CSF leak before and after the fixation surgery) were also excluded. Trauma-related CSF leak was defined as CSF leak after traumatic injury of the head or spine regions. Surgery-related CSF was considered if there was no CSF leak right after the trauma but there was after the trauma surgery. Complicated head injuries with epidural hemorrhage, subdural hemorrhage, or subarachnoid hemorrhage were excluded. Furthermore, patients with bony problems, such as osteomyelitis or cancer infiltration, were excluded as the conditions themselves can cause repetitive CSF leak [9,10].

The conservative treatment protocol included intravenous hydration, optional caffeine intake, and strict bed rest without head elevation for over 23 h a day [11]. Eating, urination, and defecation were done in bed in the lateral recumbent position. If there was visible leakage from the skin in a procedure-related CSF leak, then a water-tight skin suture was required. If clinical symptoms disappeared for 8 h, then the head would be elevated at 30 degrees for 4 h, followed by 45 and 60 degrees for 4 more hours each before being positioned fully upright. Patients would be put back lying flat once symptoms recurred.

Clinical data, imaging findings, treatment options, duration of hospitalization, and symptom recurrence were collected and analyzed. Onset duration was defined as the time period from the day of symptom onset to the day of seeking medical assistance. Conservative treatment duration refers to the time span from the day of hospitalization to the day of first invasive management or discharge. Hospitalization duration lasted from admission until discharge; for those who had CSF leak after a procedure during the same hospitalization, the hospitalization duration was counted from the day of symptom onset to discharge. Days before recurrence were calculated from the day of the last successful treatment to the day of symptom recurrence.

The two-tailed Student’s *t* test was used for continuous variables. The chi-square test was utilized for categorical data; if the data number was fewer than 5, Fisher’s exact was applied. Ordinal data, such as the visual analogue scale for pain, were analyzed with the Mann–Whitney test. Statistical significance was defined as *p* < 0.05. All statistical analyses were carried out using R (version 4.1.1) for Windows.

MRI findings of intracranial hypotension were defined as enhancement of the pachymeninges or any two of the following: subdural fluid accumulation, engorgement of venous structures, pituitary hyperemia, and sagging of the brain [12,13]. Each image was reviewed by two separate radiologists, and a third radiologist would be recruited if there was no consensus reached.

This study was approved by the E-DA Hospital Institutional Review Board (IRB)—EMRP-110-115.

## 3. Results

**Figure 1:**
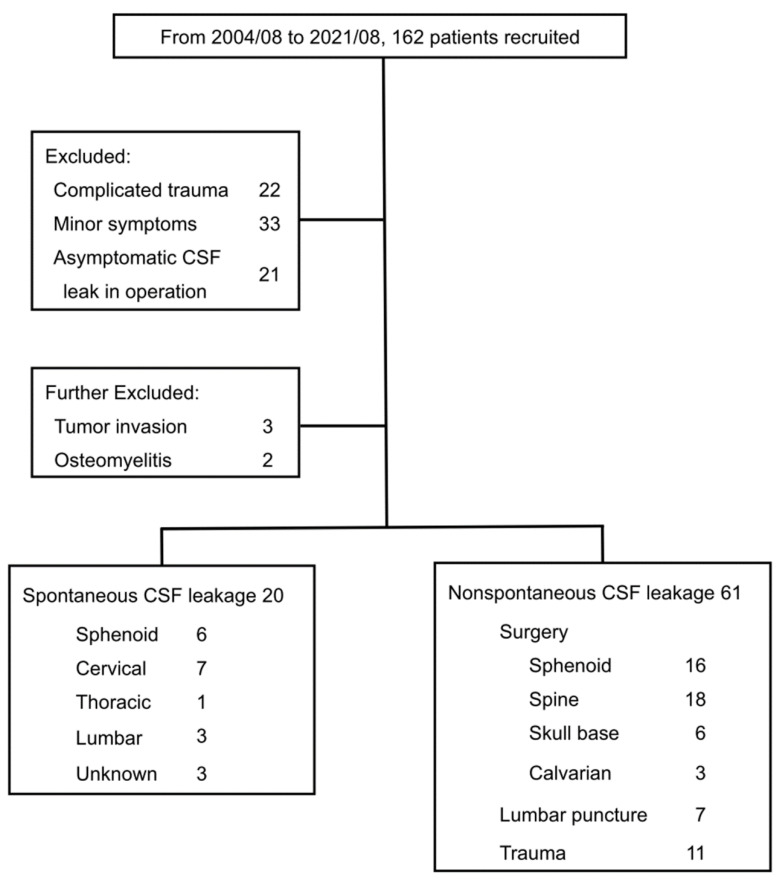
Study algorithm.

From August 2004 to August 2021, a total of 162 patients were recruited. Twenty-two patients had complicated head injuries with CSF leak before and after the surgery. Thirty-three patients had minor symptoms that did not fit the criteria of *The International Classification of Headache Disorders*, 3rd edition. Twenty-one patients had accidental CSF leak during surgery but did not have symptoms postoperatively, and all of them were excluded. We further excluded three patients with tumor invasion of the skull bone and two patients with osteomyelitis. Eight-one patients entered the final analysis (Figure 1).

The average age was 43.2 ± 13.8 and the gender ratio was 44:37 (female:male). We divided patients into two groups based on the spontaneity of leak (Table 1). The spontaneous CSF leak group had 20 patients and the nonspontaneous CSF leak group had 61 patients. There were no differences in the demographic data, including age, gender, height, body weight, and BMI (Supplementary Table S1). Spontaneous CSF leak patients had longer symptom onset durations, but the onset symptoms, the pain scale, and the percentage of meningitis showed no differences. Spontaneous CSF leak patients also had a higher proportion of MRI findings of CSF accumulation. All patients had similar treatment courses. Conservative or supportive treatments were applied first, while advanced therapy, such as blood patch or surgery, ensued if conservative treatments failed. The nonspontaneous group had longer conservative treatment durations and tended to use surgery as the advanced treatment, while the spontaneous group tended to be treated with a blood patch. Recurrence rates were similar in both groups. Once symptoms recurred, the nonspontaneous group had longer management durations. If the recurrent CSF leak did not respond to conservative treatments, the nonspontaneous group favored surgical treatment and the spontaneous group preferred blood patch.

Nonspontaneous CSF leak was caused by surgery, lumbar puncture, and trauma (Table 2). Based on the sites of operation, surgery-related CSF leak was further divided into sphenoid, spine, skull base, and calvaria. The subgroup analysis showed statistical differences in the number of repeated operations, the duration between the last procedure to symptom onset, symptom onset duration, and pain scale. Calvarian surgery required three repetitive operations on the same site before CSF leak; the other procedures required only one. Calvarian surgery had the longest duration between the procedures and symptom onset (201 ± 196 days). The symptom onset duration was the longest for spinal surgery (6.6 ± 6.5 days) and the shortest for lumbar puncture (1.9 ± 0.8 days). Spinal surgery had the highest pain score at 5.9 ± 1.5, in contrast to approximately two of the other surgeries. A detailed 2-by-2 Fisher’s exact test showed that the initial symptom of spinal surgery and lumbar puncture was only headache, while sphenoid surgery, skull-base surgery, and trauma-related CSF leak might present headache or rhinorrhea (Supplementary Table S2). One patient with skull-base surgery presented with right abducens palsy. The clinical presentation of calvarian surgery was more complicated, including headache, meningitis with fever, and conscious disturbance. Meningitis was present in 1/3 of the sphenoid, skull base, and calvarian surgeries and in 18.2% of the trauma patients. Patients who had had spinal surgery or lumbar puncture did not report having meningitis. Spinal surgery and trauma had a higher proportion of image findings of CSF accumulation on the MRIs, while skull-base surgery, calvarian surgery, and lumbar puncture did not have such MRI findings. As for the treatment, the success rates and durations of conservative treatment were similar in all groups. CSF leak related to lumbar puncture tended to use blood patch as the advanced therapy, but surgery or trauma-related CSF leak inclined toward surgical repair. The recurrence rates were similar in all groups, approximately 30%. The sphenoid surgery group had the longest management duration for recurrence (30.3 ± 5.6 days).

**Figure 2:**
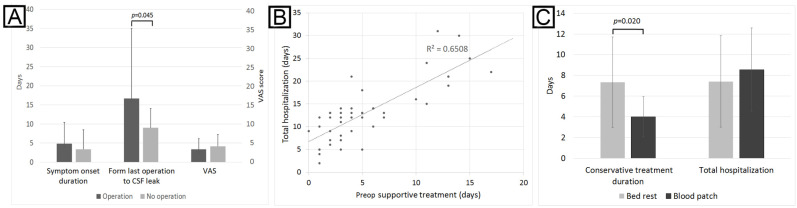
Subgroup analysis of surgery-related and spinal CSF leak. Panel (**A**): in all surgery-related CSF leak, only “interval from the last operation to CSF leak” was related to clinical decision to reoperate. Panel (**B**): in all surgery-related CSF leak, there was a strong correlation between preop supportive durations and total hospitalization duration, which indicated that earlier reoperation can shorten hospital stay. Panel (**C**): In spontaneous spinal and lumbar puncture-related CSF leak, patients had, on average, 4 days of conservative treatments before being referred for blood patch. Patients without blood patch needed an average of 7.33 days of conservative treatments. However, there was no difference in total hospital stay.

In surgery-related CSF leak that required advanced surgical treatment, further analysis showed that the surgeons’ decisions for reoperation were associated with longer intervals from the last operation to CSF leak but not with symptom onset duration or pain severity (Figure 2A). Once CSF leak happened, the preop supportive treatment duration had a good correlation with the total hospitalization days (Figure 2B), which indicated that earlier reoperation could shorten hospitalization. However, there was no difference in recurrence rates between the reoperation group (55.3%) and the conservative group (56.3%). Spinal surgery-related CSF leak included 2 cervical, 1 thoracic, and 15 lumbar spinal surgeries. Interestingly, 11 patients were operated on by orthopedics, 6 by neurosurgeons, and 1 by the general surgeon. Two were operated on by a minimally invasive procedure and 16 by an open approach. 

Trauma-related CSF leak was predominantly in males (male:female = 10:1) at the age of 34.5 ± 10.6 years old. Symptoms occurred 2 ± 1.7 days after trauma and rhinorrhea was more common than headache as the presenting symptom. Image findings of CSF collecting in the anterior skull base were found in 54.5% of the patients.

Spontaneous CSF leak was subdivided anatomically into sphenoid, cervical, thoracic, and lumbar regions based on the leaking sites (Table 3). The unknown cases refer to the patients who met the criteria of headache attributed to low CSF pressure without detectable leaking sites. Some subgroup data were not analyzed statistically because of insufficient power. Sphenoid CSF leak patients all presented with rhinorrhea and those with spinal CSF leak presented with headache. Treatment strategy was also different: 16.7% conservative treatment in sphenoid but 50% in spinal leak. With unsuccessful conservative treatments, patients with sphenoid CSF leak underwent surgery, while those with spinal CSF leak received a blood patch. The recurrence rates in both subgroups were similar.

**Figure 3:**
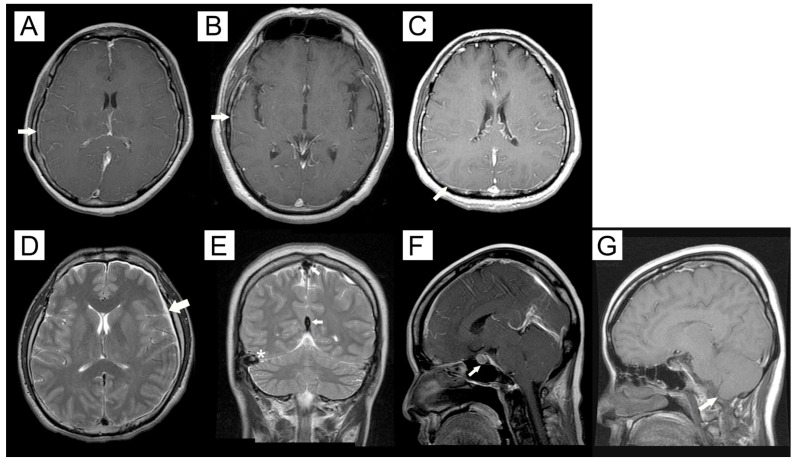
MRI findings of intracranial hypotension. Panels (**A**–**C**) were T1-weighted images with contrast enhancement (arrows). Panel (**A**) revealed generalized enhancement of the meninges, while panel (**B**) showed partial enhancement of the frontotemporal region, and panel (**C**) showed partial enhancement of the occipital region. Panel (**D**): subdural fluid accumulation on the left hemisphere (arrow) on T2-weighted image. Panel (**E**): engorgement of the superior sagittal sinus (arrows) and transverse sinus (asterisks) on T2-weighted image. Panel (**F**): T1-weighted image with contrast enhancement demonstrates pituitary hyperemia (arrow). Panel (**G**): T1-weighted image without contrast shows brain sagging with cerebellar tonsil herniation (arrow).

Seventy-nine percent of patients with spontaneous spinal CSF leak had MRI findings of both intracranial hypotension and 71.4% showed CSF accumulation. MRI findings of intracranial hypotension had an overall sensitivity of 85.7%. With detailed analyses, enhancement of the pachymeninges showed the highest sensitivity of 78.6%, followed by subdural fluid accumulation and sagging of the brain, both 21.4%, and subsequently by engorgement of venous structures and pituitary hyperemia, both 14.3%. We arrived at a new finding that enhancement of the pachymeninges was not necessary to involve the whole of the meninges. One patient had involvement of the frontotemporal lobe and the other had involvement of the occipital area only. Both were confirmed with low intracranial pressure by lumbar puncture (Figure 3).

We further analyzed the results of blood patch on spinal CSF leak, including the spontaneous spinal CSF leak group and the lumbar puncture subgroup of the nonspontaneous CSF leak patients. There were 9 patients treated with blood patch and 12 patients treated supportively. Patients treated with blood patch had shorter conservative treatment durations of 4 ± 1.9 days, compared with the 7.3 ± 4.4 days in the conservative treatment group; however, the total hospitalization showed no difference (Figure 2C). Thus, an average of 4 days of conservative treatments showed 57.1% response. There were no significant differences in MRI findings of CSF accumulation (*p* = 0.462), MRI signs of intracranial hypotension (*p* = 0.070), symptom onset durations (*p* = 0.058), or pain scale (*p* = 0.085). Interestingly, among the patients treated with blood patch, two had recurrences, and among those treated supportively, three had recurrences. The recurrence rates were similar (*p* = 1).

## 4. Discussion

This is the first systematic evaluation of the management and assessment of the outcomes of all causes of CSF leak. Previous studies mainly focused on incidence or prevention of procedure-related CSF leak. In surgery-related CSF leak, we excluded 21 patients of incidental durotomy during surgery without clinical symptoms. A previous study on spinal surgery showed that incidental durotomy was found in 3.84% of all spinal surgeries and only 1/4 of them had clinical symptoms [14]. This explained our limited case number even with 17 years of recruitment.

Spinal surgery-related CSF leak had a prevalence from 4.7% to 14% at the lumbar spine and 1% at the cervical spine. With regard to surgical procedures, cervical anterior decompression had the lowest incidence (0.42%) of CSF leak, and lumbar decompression ± fusion without instrumentation had the highest rate (17%) [14]. In lumbar spinal surgeries, minimally invasive surgery had a lower prevalence rate of CSF leak than open surgery (4.7% vs. 9.0%) [8]. Among our 15 patients of CSF leak after lumbar spinal surgery, only one was operated on by minimally invasive surgery. Although minimally invasive procedures are still not widely conducted, they have shown benefits in reducing CSF leak [15]. Interestingly, MRI fluid accumulation after minimally invasive lumbar discectomy is mainly due to saline accumulation instead of CSF leak [16]. For the management of CSF leak, 44.4% of our patients responded well to conservative treatments, similar to the previous data [17]. Ten patients (55.6%) who required surgical repair all had traditional direct suturing and only one had recurrence. Several new invasive treatments have been introduced, such as percutaneous injection of cryoprecipitate with 10% calcium [18] and blood patch [19], with promising results. Generally, CSF leak after spinal surgery had a good prognosis.

Meningitis was a common complication during the treatment. There was no difference in the prevalence of meningitis between the spontaneous and nonspontaneous groups; however, subgroup analysis of nonspontaneous CSF leak showed higher meningitis complication rates in sphenoid, skull base, and calvarian surgeries, compared with spinal surgery, trauma, and lumbar puncture. This suggests that surgeries involving the skull have a higher chance of meningitis complication.

Our findings showed that earlier reoperation would shorten the hospital stay. However, surgeons preferred conservative treatment unless there was a longer interval between the last operation and CSF leak. We further analyzed the recurrence rates of surgical and conservative treatment groups and found that there was no significant difference. Earlier reoperation could shorten the hospital stay but could not reduce the recurrence. Reoperation, of course, cost more than the conservative treatment did.

Sphenoid and spinal CSF leak were both considered as spontaneous CSF leak but they were fundamentally different. All cases of sphenoid CSF leak presented with rhinorrhea but not headache, which was similar to previous reports [12]. Sphenoid CSF leak favored surgical rather than supportive treatment. Meningitis tended to occur with sphenoid CSF leak only. In relapse patients, sphenoid CSF leak tended to have longer durations before recurrence. Sphenoid CSF was proposed to have a different pathological mechanism than spinal CSF leak. Schievink et al. reported no brain MRI manifestation of intracranial hypotension [20]. However, in our series, we saw MRI findings in all sphenoid leak patients. MRI signs were indicators of intracranial hypotension. There were some theories of intracranial hypertension at the beginning of sphenoid CSF leak, and it was reported that the CSF pressure turned to normal or low with time [21]. Our sphenoid CSF leak patients had symptom onset durations of 17 ± 11.1 days and could have a higher chance of developing hypotension. For spontaneous CSF leak without a notable leaking site, identifying clinical traits helps with the localization. Three patients in our series all presented with headache and had good responses to conservative treatment with an average hospitalization of 7.3 ± 2.6 days. Thus, we presumed that these three patients had spontaneous CSF leak in the spine rather than in the sphenoid.

In spontaneous CSF leak of the spine, although our case number was not large, the demographic data were similar to those previously reported with female predominance and onset age in the forties. MRI was reported to be diagnostic of approximately 80% of the cases, which is also similar to our findings at 85% [22]. Our data showed effectiveness of conservative treatment in 57.1% of the patients and the average conservative treatment duration was 4 days. A UK study reported a successful rate of conservative treatments at 7.8% but the duration of conservative treatments was not reported [23]. Wu et al. analyzed Taiwanese patients from a different geographic area and had patients referred for blood patch after 3 days of conservative treatment, which showed the successful rate of conservative treatments was only 8.4% [24]. Although there were some other contributing factors, our study suggested that one more day of conservative treatments may remarkably reduce the necessity of invasive treatments. The successful rate of our first attempt of blood patch was 77.8%, which was higher than the previously reported 58.7% but was lower than the 80% success rate of selective patients [24] who had anterior epidural CSF accumulation involving less than 8 segments and an injected blood volume of over 22.5 mL. We did not further analyze the MRI findings or volume of blood injected due to our small case number. However, our patients may have been mixed with both selective and general conditions. Our patients were referred for blood patch after an average of 4 days of conservative treatment, and one more day of conservative treatments may further increase the success rate of blood patch. The comparable recurrence rates between conservative treatment and blood patch in our study did not imply a lack of advantage of blood patch because blood patch may be reserved for patients who would have failed in the conservative treatments [25].

We had a strict definition of trauma-related CSF leak, which was not complicated with intracranial hemorrhage and needed to be severe enough to cause clinical symptoms. Young male with rhinorrhea was the most common presenting symptom; 81.8% required surgical treatments. A recent large-scale review of CSF leak following skull-base trauma had a similar demographic data to ours [26]. We had only two (18.2%) patients treated supportively; however, one had a symptom relapse and eventually needed surgical repair. This made a total of 90.9% trauma-related CSF leak that required surgical repair. Another retrospective study of 34 patients also had a similar finding that 27 patients initially under conservative treatments finally needed repair surgery [27]. We thus suggested trauma-related CSF leak required aggressive surgical treatments.

This study has several limitations. The case number of each subgroup was relatively small, which imposed statistical limitations on detailed subgroups analyses. We tried to determine the demographic and treatment variances in different group of patients; however, multiple comparisons further impact on the significance. This is a retrospective study and some data were not documented universally, such as the time span from the uprightness to the headache onset, the history of connective tissue disorder, and the CSF pressure. Ex post facto analysis was thus restricted. We did not have data on the total number of operations performed in each subgroup and thus the actual incidence and prevalence could not be calculated. We considered sphenoid and spinal leak together as the spontaneous CSF leak and conducted the analysis. They are significantly different in structural, pathologic, etiological, and treatment perspective. In spontaneous spinal and lumbar puncture-related CSF leak, 42.9% of the patients were referred for blood patch after conservative treatment for 4 ± 1.9 days. Thus, we conclude that, after an average of 4-day conservative treatments, 57.1% of these patients showed good responses. However, further prospective study is required to confirm our presumption.

## 5. Conclusions

We conducted a retrospective study on all causes of CSF leak. Interestingly, although patient groups were previously considered different, there were no differences in demographic data, initial symptoms, days of hospitalization, and even recurrence rates between spontaneous and nonspontaneous CSF leak. In surgery-related CSF leak, calvarian surgery needed three repetitive operations while other surgeries required just one operation. Meningitis was more common in surgery involving the skull and did not happen in spinal surgery or lumbar puncture-related CSF leak. In spontaneous CSF leak, leak from the sphenoid and from the spine showed a difference in initial symptoms and ways of management, while MRI findings and the recurrence rate were similar. Enhancement of the pachymeninges was most sensitive for intracranial hypotension on MRI. For the management of surgery-related CSF leak, earlier surgery repair was associated with a shorter hospital stay, but only longer intervals between surgery and CSF leak encouraged surgeons to reoperate. We advised that trauma-related CSF leak be treated aggressively. Spontaneous CSF leak had a 57.1% response rate under the 4-day supportive treatment. Blood patch was reserved for more severe conditions but did not shorten or prolong hospitalization.

## Figures and Tables

**Table 1:** Demographic data of spontaneous and nonspontaneous CSF leak.

	Spontaneous	Nonspontaneous	*p*-Value
Patient number	20	61	
Age	43.6 ± 9.2	43.1 ± 15.2	0.888
Gender (M:F)	6:14	31:30	0.105
Onset duration (days)	8.9 ± 9.4	3.6 ± 4.7	0.033
Headache as the first symptom	14 (70%)	29 (47.5%)	0.081
Headache pain score	5.4 ± 2.4	4.3 ± 2.1	0.093
Meningitis	2 (10%)	10 (16.4%)	0.721
MRI of CSF accumulation	16 (80%)	31 (51%)	0.035
Management			
Conservative	8 (40%)	21 (34.4%)	0.652
Blood patch	7 (35%)	2 (3.2%)	<0.001
Surgery	5 (25%)	38 (62.4%)	0.047
Conservative treatment duration (days)	6.5 ± 4.5	11.6 ± 18.1	0.048
Hospitalization (days)	10.6 ± 4.2	13.7 ± 13.2	0.109
Recurrence	8 (40%)	13 (21.3%)	0.098
Days before recurrence	58.9 ± 87.0	34.5 ± 38.4	0.517
Recurrence management Supportive	3	2	0.325
Blood patch	3	0	0.042
Surgery	2	11	0.018
Recurrence management duration (days)	9.4 ± 3.0	17.5 ± 9.3	0.02
3rd recurrence	1 (5%)	1 (1.6%)	1

**Table 2:** Subgroup analysis of nonspontaneous CSF leak.

Nonspontaneous CSF Leak							
	Sphenoid Surgery	Spine Surgery	Skull Base Surgery	Calvarial Surgery	Lumbar Puncture	Trauma	*p* Value
Patient number	16	18	6	3	7	11	
Open procedure	10 (62.5%)	15 (83.3%)	6 (100%)	3 (100%)	N/A	N/A	0.229
Number of operations	1 ± 0	1.1 ± 0.2	1.2 ± 0.4	3.7 ± 0.9	1	N/A	<0.001
From last procedure to symptom onset (days)	13 ± 20.7	18.6 ± 18.3	31.2 ± 37.8	201 ± 196	1.7 ± 0.8	N/A	<0.001
Symptom onset duration (days)	2 ± 2.3	6.6 ± 6.5	3.7 ± 4.6	4.7 ± 3.9	1.9 ± 0.8	2 ± 1.7	0.048
Headache: rhinorrhea	6:10	11:0 ^1^	1:4 ^2^	1:0 ^3^	7:0	3:8	6/15 ^5^
Headache VAS	2.2 ± 2.4	5.9 ± 1.5	0.75 ± 1.3	0	2.6 ± 0.7	2.3 ± 0.5	<0.001 ^4^
Meningitis	5 (31.25%)	0	2 (33.3%)	1 (33.3%)	0	2 (18.2%)	1/15 ^5^
Treatment							
Conservative	4 (25%)	8 (44.4%)	1 (16.7%)	1 (33.3%)	5 (71.4%)	2 (18.2%)	1/15 ^5^
Surgery	12 (75%)	10 (55.6%)	5 (83.3%)	2 (66.7%)	0	9 (81.8%)	4/15 ^5^
Blood patch	0	0	0	0	2 (28.6%)	0	1/15 ^5^
Conservative treatment duration (days)	9 ± 10.4	11.3 ± 14.8	4.2 ± 3.2	11.3 ± 5.7	4 ± 2.9	24.7 ± 31.6	0.131
Hospitalization days	21.9 ± 25.2	14.5 ± 18.5	10.3 ± 4.2	21 ± 7.3	4.6 ± 3.1	14.9 ± 7.9	0.372
MRI with CSF accumulation	6 (18.75%)	15 (83.3%)	0	0	0	6 (54.5%)	5/15 ^5^
Recurrence	3 (18.75%)	6 (33.3%)	0	0	0	4 (36.4%)	0/15 ^5^
Days before recurrence	23 ± 22.0	20.3 ± 14.9	N/A	N/A	N/A	67.5 ± 51.2	0.157
Recurrence management							0.692
Conservative	1 (33.3%)	1 (16.7%)	N/A	N/A	N/A	0	
Surgery	2 (66.7%)	5 (83.3%)	N/A	N/A	N/A	4 (100%)	
Recurrence management duration (days)	30.3 ± 5.6	10.2 ± 1.6	N/A	N/A	N/A	16.8 ± 6.4	0.002

^1^ Three patients with low back pain, two with neck pain, one with dizziness, and one with lump of the lumbar spine; ^2^ one patient with right abducens palsy; ^3^ one patient with conscious disturbance and one with fever; ^4^ Kruskal–Wallis test, calvarial surgery was excluded because of insufficient case number; ^5^ number of significant Fisher exact tests among total 15 tests. Please see supplementary data for more details. N/A: not applicable.

**Table 3:** Subgroup analysis of spontaneous CSF leak.

Spontaneous CSF Leak							
	Sphenoid	Spinal				Unknown	*p* Value *
		Cervical	Thoracic	Lumbar	Total		
Patient number	6	7	1	3	11	3	
Symptom onset (days)	17 ± 11.1	3.3 ± 2.8	4	3 ± 1	3.3 ± 2.4	14 ± 5.1	
Headache: rhinorrhea	0:6	7:0	1:0	3:0	11:0	3:0	<0.001
Headache VAS	N/A	5.1 ± 2	3	5.3 ± 3.4	5 ± 2.5	7 ± 0.8	
Meningitis	2 (33.3%)	0	0	0	0	0	0.5
Treatment							
Conservative	1 (16.7%)	3 (42.9%)	0	1 (33.3%)	4 (36.4%)	3 (100%)	0.6
Nonconservative	5 (83.3%)	4 (57.1%)	1 (100%)	2 (66.7%)	7 (63.6%)	0	
Conservative treatment duration (days)	5.6 ± 5.1	7.6 ± 5	13	5.3 ± 1.9	6.7 ± 4.3	7.3 ± 2.6	
Hospitalization days	12.8 ± 3.9	9.4 ± 4.4	13	11 ± 2.2	10.2 ± 3.9	7.3 ± 2.6	
MRI with intracranial hypotension	6 (100%)	6 (85.7%)	1 (100%)	3 (100%)	10 (91%)	1 (33.3%)	1
MRI with CSF accumulation	6 (100%)	6 (85.7%)	1 (100%)	3 (100%)	10 (91%)	0	1
Recurrence	3	2	0	2	4 (36.3%)	1	0.80
Days before recurrence	128 ± 112	22.5 ± 15.3	0	3	12 ± 15.2	30	
Recurrence management Conservative	1	1	0	0	1	1	0.786
Blood patch	0	1	0	2	3	0	0.125
Surgery	2	0	0	0	0	0	0.429
Recurrence management duration (days)	9 ± 0	9.5 ± 5.6	0	15	8.5 ± 5.7	5	
3rd recurrence	1	0	0	0	0	0	1

* Comparison between sphenoid and total spinal CSF leak.

## Data Availability

The data supporting the current study are available from the corresponding author upon reasonable request.

## Supplementary Materials

The following supporting information can be downloaded at: https://www.mdpi.com/article/10.3390/brainsci12030340/s1, Table S1: Demographic data of spontaneous and nonspontaneous CSF leak, Table S2: Detailed results of Fisher’s exact test on binomial data in Table 2.
