# Supplementary material for: Treatment, Outcome, and Relapse of Spontaneous and Nonspontaneous Cerebrospinal Fluid Leak
Source: Brain Sci. 2022 Mar 2;12(3):340. doi: 10.3390/brainsci12030340 (PMC8945904; doi:10.3390/brainsci12030340)
Supplement: Supplementary file 1 [file brainsci-12-00340-s001.zip › brainsci-1582087-supplementary.pdf]

Supplementary Materials

Table S1. Demographic data of spontaneous and non-spontaneous CSF leak.

	Spontaneous	Non-spontaneous	*p*-value
Patient number	20	61	
Height (cm)	164±66	165±63	0.36
Body weight (kg)	64.4±13.1	65.7±13.3	0.36
BMI	23.8±11.6	24.2±23.1	0.33

Table S2. Detailed results of Fisher's exact test on binominal data of Table 2

Head : rhinorrhea

	Sphenoid surgery	Spine surgery	Skull base surgery	Calvarial surgery	Lumbar puncture	Trauma
Sphenoid surgery	-	**0.0011**	0.6244	0.4118	**0.0075**	0.6924
Spine surgery	-	-	**0.0027**	1	1	**0.001**
Skull base surgery	-	-	-	0.333	**0.010**	1
Calvarial surgery	-	-	-	-	1	0.333
Lumbar	-	-	-	-	-	**0.004**

puncture						
Trauma	-	-	-	-	-	-

Meningitis

	Sphenoid surgery	Spine surgery	Skull base surgery	Calvarial surgery	Lumbar puncture	Trauma
Sphenoid surgery	-	**0.0157**	1	1	0.2719	0.6618
Spine surgery	-	-	0.0543	0.1429	1	0.1355
Skull base surgery	-	-	-	1	0.1923	0.584
Calvarial surgery	-	-	-	-	0.3	1
Lumbar puncture	-	-	-	-	-	0.4967
Trauma	-	-	-	-	-	-

Conservative

	Sphenoid surgery	Spine surgery	Skull base surgery	Calvarial surgery	Lumbar puncture	Trauma
Sphenoid surgery	-	0.2966	1	1	0.0657	1

Spine surgery	-	-	0.3509	1	0.3783	0.2344
Skull base surgery	-	-	-	1	0.1026	1
Calvarial surgery	-	-	-	-	0.5	1
Lumbar puncture	-	-	-	-	-	**0.0491**
Trauma	-	-	-	-	-	-

Surgery

	Sphenoid surgery	Spine surgery	Skull base surgery	Calvarial surgery	Lumbar puncture	Trauma
Sphenoid surgery	-	0.2966	1	1	**0.0013**	1
Spine surgery	-	-	0.3509	1	**0.0202**	0.2344
Skull base surgery	-	-	-	1	**0.0047**	1
Calvarial surgery	-	-	-	-	0.0667	1
Lumbar puncture	-	-	-	-	-	**0.0023**
Trauma	-	-	-	-	-	-

Blood patch

	Sphenoid surgery	Spine surgery	Skull base surgery	Calvarial surgery	Lumbar puncture	Trauma
Sphenoid surgery	-	1	1	1	0.083	1
Spine surgery	-	-	1	1	**0.007**	1
Skull base surgery	-	-	-	1	0.4615	1
Calvarial surgery	-	-	-	-	1	1
Lumbar puncture	-	-	-	-	-	0.1373
Trauma	-	-	-	-	-	-

MRI with CSF accumulation

	Sphenoid surgery	Spine surgery	Skull base surgery	Calvarial surgery	Lumbar puncture	Trauma
Sphenoid surgery	-	**0.0122**	0.1328	0.517	0.1243	0.4517
Spine surgery	-	-	**0.0006**	**0.015**	**0.0002**	0.1975
Skull base	-	-	-	1	1	**0.0427**

surgery						
Calvarial surgery	-	-	-	-	1	0.2088
Lumbar puncture	-	-	-	-	-	**0.0377**
Trauma	-	-	-	-	-	-

Recurrence

	Sphenoid surgery	Spine surgery	Skull base surgery	Calvarial surgery	Lumbar puncture	Trauma
Sphenoid surgery	-	0.4479	0.5325	1	0.5257	0.3919
Spine surgery	-	-	0.277	0.5263	0.1372	1
Skull base surgery	-	-	-	1	1	0.2374
Calvarial surgery	-	-	-	-	1	0.5055
Lumbar puncture	-	-	-	-	-	0.119
Trauma	-	-	-	-	-	-
